# One Health surveillance of West Nile and Usutu viruses: a repeated cross-sectional study exploring seroprevalence and endemicity in Southern France, 2016 to 2020

**DOI:** 10.2807/1560-7917.ES.2022.27.25.2200068

**Published:** 2022-06-23

**Authors:** Orianne Constant, Patricia Gil, Jonathan Barthelemy, Karine Bolloré, Vincent Foulongne, Caroline Desmetz, Agnès Leblond, Isabelle Desjardins, Sophie Pradier, Aurélien Joulié, Alain Sandoz, Rayane Amaral, Michel Boisseau, Ignace Rakotoarivony, Thierry Baldet, Albane Marie, Benoît Frances, Florence Reboul Salze, Bachirou Tinto, Philippe Van de Perre, Sara Salinas, Cécile Beck, Sylvie Lecolinet, Serafin Gutierrez, Yannick Simonin

**Affiliations:** 1 Pathogenesis and Control of Chronic and Emerging Infections, Montpellier University, INSERM, EFS (etablissement français du sang), Montpellier, France; 2 ASTRE research unit, CIRAD, INRAe, Montpellier University, Montpellier, France; 3 BioCommunication en CardioMétabolique (BC2M), Montpellier University, Montpellier, France; 4 EPIA, UMR 0346, Epidemiologie des maladies animales et zoonotiques, INRAE, VetAgro Sup, Marcy l’Etoile, France; 5 University of Lyon, VetAgro Sup, GREMERES-ICE Lyon Equine Research Center, Marcy l’Etoile, France; 6 Jolimont veterinary clinics, Toulouse, France; 7 National veterinary school of Toulouse, Université de Toulouse, Toulouse, France; 8 Aix Marseille Université – CNRS, UMR 7376, Laboratoire Chimie de l’Environnement, Marseille, France; 9 UMR 1161 Virology, ANSES, INRAE, ENVA, ANSES Animal Health Laboratory, EURL for equine diseases, Maisons-Alfort, France; 10 EID Méditérranée, Montpellier, France; 11 Veterinary Clinic des étangs, Perols, France; 12 CIRAD, UMR ASTRE, CRVC, Petit Bourg, France

**Keywords:** West Nile virus, Usutu virus, co-circulation, One Health surveillance, seroprevalence

## Abstract

**Background:**

West Nile virus (WNV) and Usutu virus (USUV), two closely related flaviviruses, mainly follow an enzootic cycle involving mosquitoes and birds, but also infect humans and other mammals. Since 2010, their epidemiological situation may have shifted from irregular epidemics to endemicity in several European regions; this requires confirmation, as it could have implications for risk assessment and surveillance strategies.

**Aim:**

To explore the seroprevalence in animals and humans and potential endemicity of WNV and USUV in Southern France, given a long history of WNV outbreaks and the only severe human USUV case in France in this region.

**Methods:**

We evaluated the prevalence of WNV and USUV in a repeated cross-sectional study by serological and molecular analyses of human, dog, horse, bird and mosquito samples in the Camargue area, including the city of Montpellier, between 2016 and 2020.

**Results:**

We observed the active transmission of both viruses and higher USUV prevalence in humans, dogs, birds and mosquitoes, while WNV prevalence was higher in horses. In 500 human samples, 15 were positive for USUV and 6 for WNV. Genetic data showed that the same lineages, WNV lineage 1a and USUV lineage Africa 3, were found in mosquitoes in 2015, 2018 and 2020.

**Conclusion:**

These findings support existing literature suggesting endemisation in the study region and contribute to a better understanding of USUV and WNV circulation in Southern France. Our study underlines the importance of a One Health approach for the surveillance of these viruses.

## Introduction

West Nile virus (WNV) and Usutu virus (USUV) are neurotropic mosquito-borne flaviviruses belonging to the Japanese encephalitis serocomplex [1]. Circulation of these viruses is maintained in similar transmission cycles involving ornithophilic mosquitoes, such as *Culex pipiens*, and different bird species. Both viruses can also spread to several mammalian species, including humans, although most mammals are considered to be dead-end hosts [2]. WNV can represent a serious burden to human and animal health [3]. The clinical spectrum of WNV infection in humans can range from influenza-like febrile illness to acute flaccid paralysis, meningitis or encephalitis [4]. USUV has been shown to affect primarily specific species of wild birds (mainly blackbirds and owls in Europe) [5,6]. Although less numerous than human cases of WNV infections, USUV infections in humans can manifest as encephalitis, meningitis and meningoencephalitis in both immunocompetent and immunocompromised patients [5].

Despite their potential impact on human and animal health, the available measures to control these viruses are limited. Although inactivated and recombinant WNV vaccines exist for horses [7], there are currently no specific treatments or vaccines for WNV or USUV available for humans. The management of virus circulation is thus largely based on integrated surveillance activities. These activities provide guidance to implement mainly preventive measures in case of detection. For example, blood for donation is tested for WNV and donors are deferred in regions in which outbreaks are detected because WNV can be transmitted through blood transfusion [8].

Since the 1960’s, different lineages of WNV and USUV have been periodically detected in Europe. These viruses were mainly associated with sporadic cases in humans, horses and birds and limited to certain European regions, until recently [9]. Occasional outbreaks of WNV have mainly taken place in Southern Europe during the last two decades [10-12]. Nevertheless, the outbreaks of both viruses have recently increased in numbers and geographical distribution. In 2018, a concurrent WNV and USUV epidemic occurred for the first time in several parts of Europe, including countries in different latitudes and a record number of outbreaks [2,5,13-18]. Currently, epidemiological patterns in several European regions suggest endemicity of these viruses instead of periodic introductions from endemic regions. For example, repeated detection of the same strains in the same region over a consistent number of years has been proven in Northern Italy, Hungary and Austria [19-21]. These patterns, together with the potential health risks associated with WNV and USUV, support the need for continuous rigorous analysis of the epidemiological situation in Europe [3], which will serve as a key piece of information in the design of surveillance strategies for WNV and USUV. 

This study explores the seroprevalence and potential endemicity of WNV and USUV in a European region with a high risk of endemisation. This region in the south of France includes the Rhône River delta, the French region with the longest record of WNV outbreaks and included in the French surveillance programme for WNV [9,22]. 

## Methods

### Study setting

The study area encompasses part of the Camargue area including the city of Montpellier, located within the Mediterranean basin in the eastern part of the Occitania region in Southern France. It has a climate catalogued as a warm-summer Mediterranean climate according to the Köppen-Geiger climate classification [23]. The Camargue area is a natural wetland located south of Arles city and within a triangle defined by the two arms of the Rhône River and the Mediterranean Sea. This area harbours diversified environments in which dry areas are continuously irrigated by canals, ditches and wetlands favourable to a large and diverse bird population as well as a large mosquito population [24].

### Sample collection

Sample collection was a joint effort by INSERM (French Institute of Health and Medical Research), ANSES (French Agency for Food, Environmental and Occupational Health and Safety), and CIRAD (French Agricultural Research Centre for International Development). The analyses on humans, dogs and birds were carried out by the Pathogenesis and Control of Chronic and Emerging Infections unit, INSERM, the studies on horses by the Animal Health Laboratory, ANSES and on mosquitoes by the Astre unit, CIRAD.

#### Human samples

Five hundred human serum samples randomly collected in 2019 and 2020 from outpatients attending Montpellier University hospitals (mainly for occupational medicine (n = 133) or consultations in haematology (n = 84), nephrology (n = 78), hepato-gastroenterology (n = 56), cardiology (n = 41), dermatology (n = 28)) as part of a seroepidemiological investigation. Serum samples were stored at −80^◦^C until further serological analysis. Patients less than 1 year of age and patients under care for any other viral infection were excluded from this study. An additional 34 serum samples from inpatients diagnosed in the same hospitals with non-bacterial meningitis or meningoencephalitis without an identified aetiology were also screened for WNV and USUV infection.

#### Dog samples

Serum samples were collected from 184 dogs (mainly indoor pets) in 2019–20 from animals undergoing health evaluations or surgical interventions at two veterinary clinics which serve animals from across the Montpellier area. These animals did not have any recent recorded infectious episodes. Serum samples were stored at −20 °C until processed. No animals were sampled solely for the purpose of this study.

#### Horse samples

**Figure 1:**
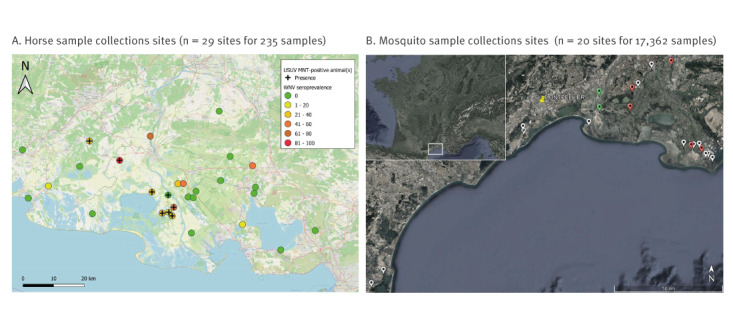
Collection sites of horse (May–June 2016; n = 29 sites) and mosquito samples (2018–2020; n = 20 sites), Southern France

MNT: microneutralisation test; WNV: West Nile virus; USUV: Usutu virus.

Serum samples from 235 horses included for the study were collected from 29 stables located in the Camargue area in May and June 2016 (Figure 1). The horse stables were chosen according to the criteria presented in Desjardins et al [25]. Samples were stored at −20°C before serological analysis. All horses were asymptomatic at the time of sampling and unvaccinated against WNV. The surveyed stables were in part randomly selected from stables located in a large area affected by WNV outbreaks in 2015. Stables that had previously reported horses with chronic fever during the sampling period or the previous year were included when possible.

A. Horse sample collections sites in the Camargue area in 2016. Stables with at least one USUV MNT-positive horse are represented by a cross and WNV seroprevalence in sampled stables is represented by a colour gradient from green (no WNV MNT-positive animal in the corresponding stable) to dark red (81–100% seroprevalence rate) (Map source: https://www.openstreetmap.org).

B. Mosquito collection (*Culex pipiens*) sites in the Camargue area from 2018 to 2020. Red pins indicate USUV-positive pools and green pins both WNV-positive and USUV-positive pools. White pins negative trapping sites. USUV-positive sites in our previous study in 2015 are shown for the sake of completeness. The location of the city of Montpellier is provided to facilitate site positioning.

The locations of sample collection for dogs and humans are not available because of the anonymisation of the data. We do not have information on the exact site where the dying wild birds were collected.

In addition to sample collection, the owner completed a questionnaire on the individual characteristics of each horse (age, sex, activity), its health status (clinical signs, infection history and treatments) and stable management (contact with other animals, tick and flea control).

#### Bird samples

From February 2019 to December 2020, sick or dying resident wild birds were passively collected around Montpellier by the Regional Centre for the Safeguarding of Wild Fauna, the League for the Protection of Birds (LPO) and Montpellier Zoological Garden for first aid care. In agreement with the competent Official Veterinary Service, samples (liver and brain) were collected from dead birds (n = 191) and were stored at −20°C for laboratory molecular investigations. 

#### Mosquito samples

Adult mosquitoes were collected from 20 sites in the Camargue area from 2018 to 2020 (Figure 1; detailed information on mosquito numbers and sites can be found in Supplementary Table S1). Overnight trapping using CO_2_-baited type traps was performed once per week between April to October (28 trappings per year), as previously described [26]. Mosquitoes were identified based on morphological features on a chill table at 4°C [26]. Once identified, non-blood-engorged *C. pipiens* females were grouped in pools of between one and 44 individuals per site and collection date and stored at −80°C until further analyses.

### Serological testing in human, dog and horse samples

A competitive enzyme-linked immunosorbent assay (cELISA, ID Screen West Nile Competition Multi species ELISA kit, IDvet, France) was used for the detection of flavivirus antibodies (IgG and IgM) in sera [27] from humans, dogs and horses, according to the manufacturer’s instructions.

To confirm WNV or USUV infection, viral microneutralisation tests (MNT) were performed on cELISA-positive sera. For human and canine samples, methods to identify USUV (France2018, MT863562) and WNV (lineage 2, MT863560) were performed according to Constant et al. [9]. For horse samples, tests using USUV (France2018, MT863562) and WNV (lineage 1IS-98-ST1, AF481864.1) were performed according to Beck et al. [28]. Different WNV strains have been used based upon their availability in the screening laboratories. WNV MNT performed on horse samples at ANSES in the frame of interlaboratory proficiency testing generated comparable results (data not shown). Briefly, serial dilutions of Dulbecco’s Modified Eagle’s Medium (DMEM) supplemented with 2% foetal bovine serum were mixed with USUV and WNV suspension at 100 tissue culture infectious dose 50 (TCID50). After incubation for 90 min at 37°C allowing for viral neutralisation, 100 µL containing 2,000 Vero cells (human samples) or 2x10^4^ Vero cells (horse samples) were mixed in each well and plates were incubated for 3 to 5 days at 37°C. The presence or absence of a cytopathic effect was determined and compared with controls. A titre was determined for each sample from the reciprocal of each dilution. The tests were performed twice in two independent experiments. For a neutralisation dilution ≥ 1:10, sera were confirmed positive for WNV or USUV antibodies after duplication of results. If the results indicate a titre for both viruses, the neutralising immune response was considered specific for WNV or USUV if the MNT titre was at least fourfold higher than the titre obtained against the other virus.

### Viral RNA detection in bird, human and mosquito samples and sequencing in mosquitoes

Viral RNA was extracted from 20–30 mg of homogenised bird brain or liver samples using the QIAmp Viral RNA mini kit (Qiagen). Viral RNA was extracted from 50 μL of human serum using the EZ1 BioRobot workstation in combination with the EZ1 DSP Virus Kit (Qiagen). The LightCycler 480 real-time PCR machine was then used for one-step reverse transcription-quantitative PCR (RT-qPCR) analysis. Mosquito pools were homogenised in PBS buffer and total RNA was extracted with the NucleoMagVet kit (Macherey Nagel) using a Kingfisher Flex (Thermo Scientific).

USUV and WNV detection was performed with a TaqMan-based RT-qPCR and a SYBR-Green-based RT-qPCR respectively, as previously described [29,30]. PCR amplification for Sanger sequencing (Eurofins Genomics, Germany) was performed using cDNA from all virus-positive pools. For USUV sequencing, we performed a PCR with the primers 4723fwd (5’-GTCATGTATGAAGGRGTT-3’) and 5672rev (5’-GTGTAYTCTGTTATCCACTC-3’). The primers target a region of the NS3 gene (their 5’ position is indicated in the name). Sequences of WNV were obtained using a PCR which targets a part of the NS5 gene as previously described to discriminate WNV lineages [31]. The GenBank accession numbers of the USUV sequences are ON125263, ON099437, ON099438, ON099439, and ON099436 for WNV sequence.

### Virus isolation

Confluent monolayers of Vero E6 (African green monkey) cells were inoculated with organ homogenates from RT-qPCR-positive birds in order to attempt virus isolation. Cells were incubated at 37°C with 5% CO_2_ for 7 days and observed daily to assess cytopathic effects.

### Phylogenetic analyses

Phylogenetic analyses were performed in Geneious (Dotmatics). Multiple alignments were generated with the default Geneious algorithm. All positions containing gaps and missing data were not included. The alignment for USUV included 33 partial sequences from mosquitoes of the NS3 gene (682 bp, positions 4,736–5,614). The alignment for WNV included 51 partial sequences (all from mosquitoes, except one from a horse) of the NS5 gene (1,000 bp, positions 9,039–10,039). Trees were generated with the Tree Builder function using the maximum likelihood approach and the Jukes-Cantor model.

### Statistical analyses 

Seroprevalence for USUV and WNV was estimated by dividing the number of positive subjects by the total number of subjects tested with two-sided exact binomial 95% confidence intervals (CI). Maximum likelihood estimates of infection rates in *C. pipiens* populations and their 95% CI were obtained with the R package binGroup.

## Results

### WNV and USUV seroprevalence and infections in humans

The human cohort included 272 (54.4%) females (median age: 43 years; interquartile range (IQR): 29–58 years) and 228 (45.6%) males (median age: 49 years; IQR: 33–68 years). We identified antibodies (IgG and IgM) against flaviviruses in 53 of the 500 samples (10.3%; 95% CI: 7.9–13.3). 

All cELISA-positive human samples were then tested using MNT against WNV and USUV (Table 1). Among these 53 samples, 15 tested positive for USUV-specific antibodies (3%; 95% CI: 1.5–4.5) and six for WNV-specific antibodies (1.2%; 95% CI: 0.25–2.15). Although antibody cross-reactions could not be ruled out, samples with equal neutralising activity for WNV and USUV (< fourfold difference in MNT titre) were considered as potential co-infections. One sample demonstrated neutralising activity for both WNV and USUV. We did not detect WNV or USUV RNA in the set of 500 human samples. 

**Table 1:** Screening for Usutu and West Nile viruses in flavivirus-positive human samples using a microneutralisation test, Southern France, 2019–2020 (n = 53)

Samples (n)	USUV MNT titre	WNV MNT titre	Infection
33	ND	ND	UD
2	10	ND	USUV
9	20	ND	USUV
3	40	ND	USUV
3	ND	20	WNV
1	ND	40	WNV
1	10	160	WVN
1	20	20	WNV and USUV
Positive samples (n)	15	6	14 USUV, 5 WNV, 1 USUV/WNV

MNT: microneutralisation test; ND: not detected; UD: undetermined; WNV: West Nile virus; USUV: Usutu virus.

A titre was determined for each sample from the reciprocal of each dilution. If the results indicate a titre for both viruses, the neutralising immune response was considered specific for WNV or USUV if the MNT titre was at least fourfold higher than the titre obtained against the other virus.

We also analysed 34 serum samples from patients diagnosed with meningitis or meningoencephalitis (of unidentified aetiology). WNV or USUV were not detected in any of these samples by serological screening or RT-qPCR.

### WNV and USUV infections in dogs

We confirmed the presence of flavivirus antibodies (IgG and IgM) by cELISA in three of the 184 dog samples analysed (1.63%; 95% CI: 0.00–3.46): two USUV antibody-positive samples were detected by MNT (1.08%; 95% CI: 0.00–2.58). One dog was positive for WNV antibodies (0.54%; 95% CI: 0.00–1.60) (Table 2). We found that USUV seroprevalence in dogs appears higher than that of WNV, although the number of positive samples was too low to infer a statistically significant pattern.

**Table 2:** Screening for Usutu and West Nile viruses in flavivirus-positive dog samples using a microneutralisation test, Southern France, 2019–2020 (n = 3)

Breed	USUV MNT titre	WNV MNT titre	Infection
German wirehaired pointer	10	ND	USUV
Jack Russell terrier	40	ND	USUV
German shepherd	ND	160	WNV
Positive samples (n)	2	1	2 USUV, 1 WNV

MNT: microneutralisation test; ND: not detected; WNV: West Nile virus; USUV: Usutu virus.

A titre was determined for each sample from the reciprocal of each dilution.

### WNV and USUV infections in horses

All horses that were sampled were asymptomatic on the date of sampling as well as during the previous WNV transmission season. Two horses that belonged to two different stables had developed a WNV infection in 2015. The sampling was performed in spring 2016, outside of the WNV transmission period in France. Most of the surveyed horses had no history of clinical signs indicative of higher risks of infection by WNV and/or USUV. We identified antibodies against flaviviruses (IgM and IgG) in 40 out of 235 sera analysed (17.02%; 95% CI: 12.21–21.82) (Figure 1) (Table 3). Among these 40 samples, nine were positive for USUV-specific antibodies (3.83%; 95% CI: 1.37–6.28) and 31 for WNV-specific antibodies (13.19%; 95% CI: 8.86–17.51) which suggests that, based on the confidence intervals, WNV seroprevalence appears to be higher than USUV seroprevalence in horses. Among these positive samples, we identified five potentially co-infected samples demonstrating neutralising activity for both WNV and USUV. Moreover, a specific flavivirus species could not be determined in five horse sera.

**Table 3:** Screening for Usutu and West Nile viruses in flavivirus-positive horse samples using microneutralisation tests, Southern France, June to May 2016 (n = 40)

Samples (n)	USUV MNT titre	WNV MNT titre	Infection
5	ND	ND	UD
2	10	ND	USUV
2	20	ND	USUV
6	ND	20	WNV
3	ND	40	WNV
1	10	80	WNV
5	ND	80	WNV
2	10	160	WNV
3	ND	160	WNV
1	10	320	WNV
2	20	320	WNV
3	ND	320	WNV
1	10	20	WNV and USUV
1	80	80	WNV and USUV
3	80	40	WNV and USUV
Positive samples (n)	9	31	4 USUV, 26 WNV, 5 USUV/WNV

MNT: microneutralisation test; ND: not detected; UD: undetermined; WNV: West Nile virus; USUV: Usutu virus. A titre was determined for each sample from the reciprocal of each dilution. If the results indicate a titre for both viruses, the neutralising immune response was considered specific for WNV or USUV if the MNT titre was at least fourfold higher than the titre obtained against the other virus.

### WNV and USUV infections in birds

Brain or liver samples from diseased, euthanised or dead-found wild birds, with or without neurological signs, were analysed for the detection of WNV and USUV RNA by RT-qPCR. We did not detect WNV RNA in any of the samples, whereas five wild birds were positive for USUV RNA (Table 4). In 2019, USUV-infected birds found included one *Columba livia domestica* (Domestic pigeon), one *Streptopelia decaocto* (Eurasian collared dove), one *Accipiter nisus* (Eurasian sparrowhawk) and one *Turdus philomelos* (Song thrush). In 2020, we identified one *Strix aluco* (Tawny owl), a species considered as highly sedentary. USUV isolation from avian samples was not successful.

**Table 4:** Wild bird species positive for Usutu virus by RT-qPCR, Southern France, 2019–2020 (n = 5)

Species	Collection date	Neurological symptoms	USUV Cq values
Domestic pigeon	23 Aug 2019	No	35.95
Eurasian collared dove	29 Oct 2019	Yes	34.59
Eurasian sparrowhawk	16 Nov 2019	No	33.65
Song thrush	7 Oct 2019	Yes	32.89
Tawny owl	10 Jul 2019	Yes	37.94

Cq: cycle quantification; USUV: Usutu virus.

Samples (brains or livers) of dying wild birds with and without neurological symptoms were analysed by RT-qPCR. No samples positive for WNV were detected.

### WNV and USUV infections in mosquitoes

A total of 17,362 non-engorged *C. pipiens* females were collected in the south of France between 2018 and 2020 and separated in 1,771 pools (Figure 1; detailed information on mosquito pool number and screening results can be found in Supplementary Table S1). All pools were analysed for the presence of WNV and USUV RNA. Two WNV-positive pools were identified in 2018, each in a different site. USUV-positive pools were detected in 2018 (one pool) and 2020 (six pools from four sites). No WNV- or USUV-positive pools were detected in 2019. The number of positive pools implied low infection rates in *C. pipiens* populations across all years, i.e. < 0.5% (infection rates available in Supplementary Figure S1). 

**Figure 2:**
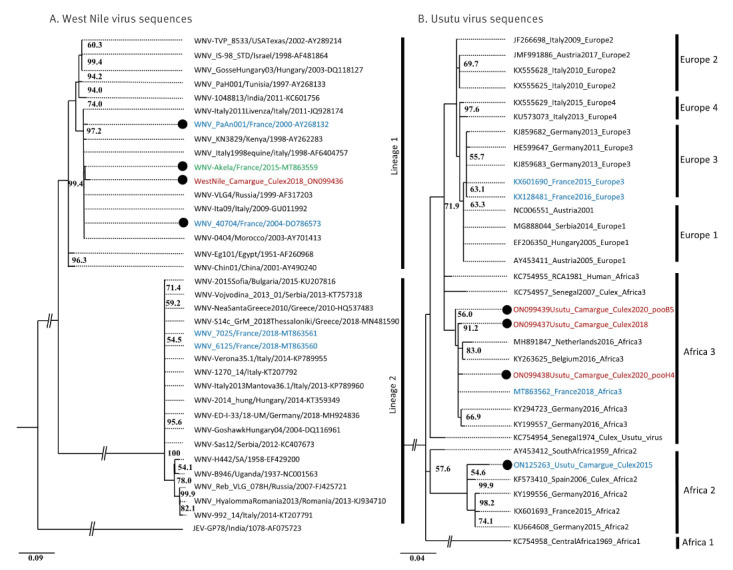
Maximum likelihood phylogenetic trees of A. the West Nile virus sequence from 2018 in Southern France (n = 1) and B. Usutu virus sequences from 2018 and 2020 in Southern France (n = 3), compared with selected sequences from GenBank (n = 65)

WNV: West Nile virus; USUV: Usutu virus.

We obtained one sequence from a WNV-positive pool in 2018 and three sequences from USUV-positive pools, one in 2018 and two in 2020 (Figure 2). The WNV sequence belonged to Lineage 1 and clustered with WNV sequences previously identified in the Camargue area isolated from horses and one bird, which were obtained in 2000, 2004 and 2015. All USUV sequences clustered together within USUV Africa 3 Lineage.

The phylogenies were inferred based on a partial sequence of the NS5 or the NS3 genes for WNV and USUV, respectively. The tree was rooted with a sequence of Japanese encephalitis virus (AF075723) for WNV sequences. The tree was rooted with a sequence of lineage Africa 1 for USUV. One hundred bootstrap datasets with random sequence addition were computed to generate a consensus tree drawn. Numbers along branches are bootstrap values. Scale bar indicates substitutions per site.

Sequences in blue come from France. French sequences identified in this study (red) are all from mosquito samples and are available in GenBank. The WNV sequence from a horse from the Camargue region in 2015 and obtained by other authors appears in green to facilitate comparison [32]. Sequences from the Camargue area are marked with a black circumference. The year of collection is stated on the sequence name, generally after the country name.

## Discussion

Here, we analysed the epidemiological situation of WNV and USUV in the Camargue area in France, a European region in which endemicity of both viruses is suspected. This seroprevalence study targeted several epidemiologically relevant hosts: humans, horses, wild birds and mosquitoes. Very low WNV seroprevalence in horses was evident in the Camargue region between 2005 and 2010 when important sampling efforts in wild birds were employed; no spillover outside the enzootic bird–mosquito transmission cycle was evident during the same period [32]. Furthermore, there is no molecular study showing the circulation of the virus over this period. WNV re-emerged in the Camargue during the summer of 2015 [32]. This arbovirus has been repeatedly reported in birds [33], horses [10,32,34,35] and humans [36-38] in this area since that time. Similarly, USUV infections have been previously reported in mosquitoes [26] and birds [33], and we previously described a human case of idiopathic facial paralysis in a person infected with USUV in the same area [39]. Although dogs do not commonly develop clinical signs, especially neurological disorders, with USUV or WNV infections, they were included in the study because they are considered relevant candidates for surveillance because of their proximity to humans and high WNV exposure rates, as previously documented [40]. Importantly, prevalence was estimated for 3 consecutive years in mosquito populations to provide a time window large enough to assess long-term virus presence. Thus, our approach aimed to obtain an estimation of virus prevalence in the study area, as well as genomic sequences over several years. Our results show that infections of USUV and WNV among humans, dogs, horses, birds and mosquitoes repeatedly occurred in the study area in recent years.

In comparison with our mosquito surveillance data from 2015 [26], we identified two sites with WNV and USUV detection in different years, suggesting a high suitability of those sites for future surveillance studies (Supplementary Table S1: mosquito collection sites). Moreover, the combination of genetic data from other studies and ours show the presence of the same lineages for both WNV and USUV in the same region in 2015, 2018 and 2020. More precisely, the cluster of WNV Lineage 1 found in mosquitoes in 2018 has also been detected in Camargue in a horse in 2015 (Figure 2A) and in mosquito excreta in 2020 (a different genomic region than that used in our analysis, GenBank accession OK489805.1 [41]). The same cluster had been detected in equines in the region in 2000 and 2004 (Figure 2A). Thus, after an 11-year period without detection, viruses belonging to the same cluster within WNV Lineage 1 have been repeatedly detected since 2015 in both vertebrates and mosquitoes. Similarly, the same USUV lineage was detected in mosquitoes from Camargue in 2015 (a sequence from a different genomic region than that used in our analysis was obtained) [26]. Thus, similarly to WNV, USUV Africa 3 Lineage has been detected in *C. pipiens* populations in Camargue in 2015, 2018 and 2020.

The repeated detection of the same lineages over several years is notable, given the usual low prevalence of these viruses, the detection of other lineages in the study area [26] and the lack of mosquito surveillance data in 2016 and 2017. Thus, the genetic data support a scenario of endemisation for both viruses in the study area. Our data also suggest that human exposure to WNV and USUV has been non-negligible in the study area (3% (95% CI: 1.5–4.5) for USUV and 1.2% (95% CI: 0.25–2.15) for WNV). Although the human cohort analysed was relatively limited in size, the seroprevalence data from domestic dogs also support active transmission of both viruses near humans. Overall, the potential continuous presence of WNV and USUV and the exposure levels observed suggest that the infection risk in the region deserves thorough characterisation. In particular, potential endemisation requires further validation through long-term studies because WNV epidemiological patterns have been shown to be complex. For example, lineage 1 of WNV was detected in the study region in 2000 and 2004 but later remained undetected until 2015. This pattern could be explained by two contrasting scenarios: extinction after introductions or, alternatively, circulation below detection thresholds for 11 years.

Our study reports the extent of USUV prevalence in the region in humans, dogs, horses, birds and mosquitoes. USUV remains largely unstudied worldwide, although the recent avian outbreaks, together with the detection of more than 100 human cases in Europe since 2008, have triggered studies on different aspects of USUV biology [5]. Our results show that the prevalence of USUV in humans may be at least as high as that of WNV, if not higher, in the study area. This observation concurs with a previous report showing a higher prevalence of USUV than WNV in zoo animals in the same area [9]. Similarly, USUV exposure in humans has been shown to be higher than that of WNV in some Italian regions [5,42-44]. Moreover, our passive surveillance in wild birds found USUV-positive birds in sedentary species, which favours long-term virus circulation locally. Mosquito surveillance data suggest that USUV can reach relatively notable prevalence levels in the region, i.e. the infection rate in mosquitoes was above 1% in late 2015. Although USUV has not been found responsible for a large number of detected human clinical cases in the study area, our results suggest that this situation could change, should a more pathogenic strain emerge, especially because USUV is described as having lineage-dependent virulence [45].

Our study provides data on the seroprevalence of WNV and USUV in different vertebrates that can be used to assess their possible interest for inclusion in surveillance schemes. Horses are considered as valuable sentinels of the risk of WNV epizootic transmission in Europe [46,47]. Based on the high seroprevalence in horses obtained in our study (13.19% for WNV and 3.83% for USUV), their suitability for USUV surveillance warrants an experimental design allowing a robust comparison of USUV seroprevalence in domestic animals. However, we did not collect horse samples from the same years as the human and dog samples, hampering such a robust comparison. Nevertheless, WNV prevalence was almost 10-fold higher in horses than in humans and dogs, and USUV prevalence in horses was at least as high as in the other hosts. 

This study also highlights the need for reinforcement of monitoring and surveillance programmes for USUV and WNV infection over several years, including monitoring of geographic spread and the dynamics of USUV and WNV transmission in both primary and incidental hosts. Such programs exist in various European countries, particularly in southern Europe, but they are mostly focused on seasonal surveillance of WNV [48-50]. However, some countries, such as Italy, have more recently expanded their programs for both viruses planned annually under the National Integrated Surveillance Plan (NISP), based on entomological, veterinary and human surveillance activities. These programmes should also involve different support actions, such as raising awareness as well as encouraging and training clinical veterinarians to better prevent, detect and alert on these infections. Nevertheless, such analysis is challenging because of several aspects of the epidemiology of WNV and USUV. First, their epidemics are not homogeneous over Europe, with certain regions experiencing concentrated outbreaks. Secondly, a robust assessment of their prevalence requires One Health, i.e. integrated surveillance in their different hosts and, thus, sampling approaches adapted to humans, equines, wild birds or mosquitoes. In addition, virus dispersal through bird movement hinders the ability to distinguish between a continuous virus presence in a given region, i.e. endemicity, and repeated virus introductions followed by extinctions. Disentangling between the two scenarios requires long-term studies showing virus prevalence in the same region over several years.

Our study had several limitations. A larger study with more samples per host species over several years would be needed to more accurately assess the infection rates of these two viruses in the different species studied. Furthermore, compared with active surveillance (entomological or on animal sentinels), passive surveillance systems (on dead birds or on diseased humans) are simpler and cheaper to implement, but the drawback lies in the inability to detect the virus early before human cases occur. A limitation of passive surveillance in birds is that not all species die from infection. In our study, only birds that died from infection or another cause were collected for study.

## Conclusions

Our results show an active spread of USUV and WNV in a particularly exposed area of France in humans, dogs, horses, birds, and mosquitoes. Overall, improving surveillance programmes for USUV and WNV as part of a One Health approach would help to anticipate the veterinary and public health risks associated with these viruses.

## Supplementary Data

483000 Supplement
