# Author's correction for Euro Surveill. 2022;27(25)

**DOI:** 10.2807/1560-7917.ES.2022.27.27.2200068c

**Published:** 2022-07-07

**Authors:** Eurosurveillance editorial team

**Affiliations:** 1 European Centre for Disease Prevention and Control (ECDC), Stockholm, Sweden

In the article ‘One Health surveillance of West Nile and Usutu viruses: a repeated cross-sectional study exploring seroprevalence and endemicity in Southern France, 2016 to 2020’ by Constant et al., published on 23 June 2022, the name of author Sylvie Lecollinet was spelled incorrectly. This was corrected on 5 July 2022 at the request of the authors.

