# Supplementary material for: One Health surveillance of West Nile and Usutu viruses: a repeated cross-sectional study exploring seroprevalence and endemicity in Southern France, 2016 to 2020
Source: Euro Surveill. 2022 Jun 23;27(25):2200068. doi: 10.2807/1560-7917.ES.2022.27.25.2200068 (PMC9229194; doi:10.2807/1560-7917.ES.2022.27.25.2200068)
Supplement: Supplement [file 22-00068_SIMONIN_Supplement.pdf]

Supplementary material

"This supplementary material is hosted by Eurosurveillance as supporting information alongside the article [One Health surveillance of West Nile and Usutu viruses: a repeated cross-sectional study exploring seroprevalence and endemicity in Southern France, 2016 to 2020], on behalf of the authors, who remain responsible for the accuracy and appropriateness of the content. The same standards for ethics, copyright, attributions and permissions as for the article apply. Supplements are not edited by Eurosurveillance and the journal is not responsible for the maintenance of any links or email addresses provided therein."

Supplemental Figure 1. Temporal dynamics of the infection rate of Usutu virus in Culex pipiens females.

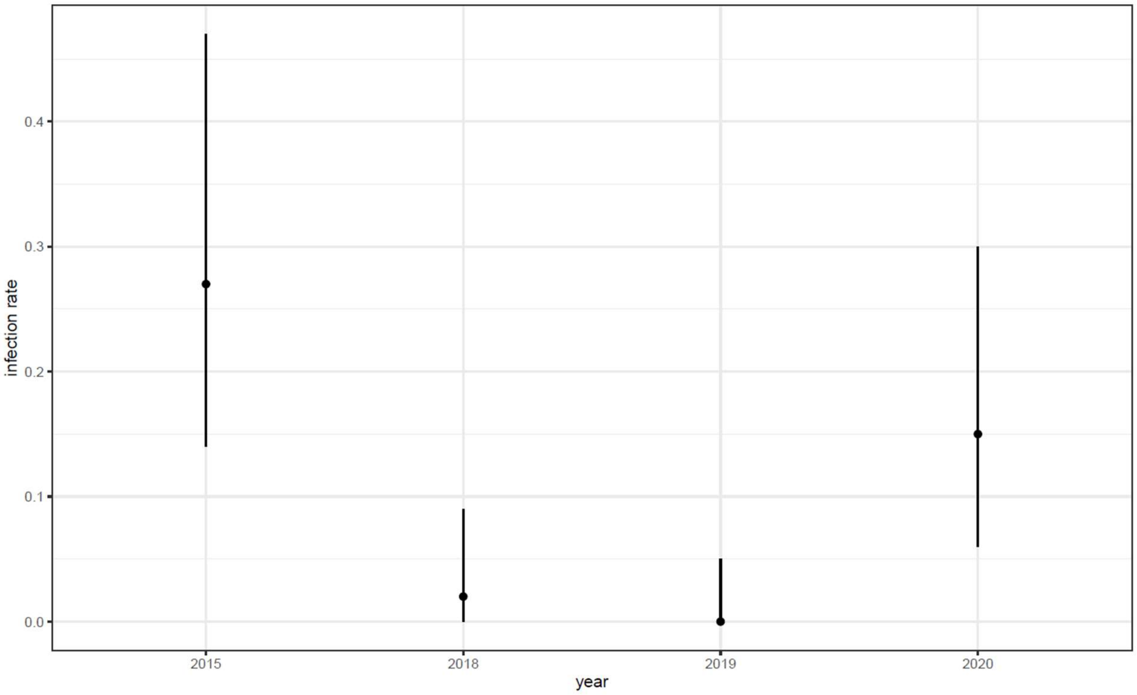

Maximum likelihood estimates of infection rates (proportion of infected females per 100 females). Estimates were obtained by pooling data from all sites for a given year.

Supplemental Table 1.

Mosquito collection sites in the Camargue area between 2018 to 2020.

sites	latitude	longitude	2015	2018	2019	2020
Beaucaire	43°45'26.48"N	4°37'0.92"E	NO	NO	YES	YES
Bellegarde	43°44'35.84"N	4°30'17.68"E	NO	NO	YES	YES
Bélugue	43°25'20.64"N	4°41'0.90"E	NO	NO	YES	YES
Bois d'Espeyran	43°38'56.88"N	4°23'36.91"E	YES	YES	YES	YES
Bois François	43°23'54.35"N	4°47'13.54"E	YES	YES	YES	YES
Caisse d'Epargne	43°24'45.84"N	4°43'38.49"E	NO	YES	YES	YES
Camping Europe	43°29'21.52"N	3°46'37.64"E	NO	NO	YES	NO
Elysette	43°30'57.87"N	4° 9'20.48"E	YES	YES	YES	YES
Eysselle	43°24'55.74"N	4°46'11.90"E	NO	NO	YES	YES
Grande Sagne	42°50'33.15"N	2°56'0.02"E	NO	YES	NO	NO
Jardins	43°24'52.78"N	4°44'30.05"E	NO	YES	YES	YES
Jasse brulee	43°37'58.24"N	4°12'22.59"E	YES	YES	YES	YES
Désert	43°28'17.94"N	3°46'49.75"E	NO	YES	YES	YES
Mas d'Avon	43°34'5.14"N	4°12'29.24"E	YES	YES	YES	YES
La Palissade	43°22'34.76"N	4°48'35.81"E	NO	NO	YES	YES
Pêcheurs	42°53'52.44"N	3° 0'0.71"E	NO	YES	NO	NO
Petite Sylve	43°34'16.56"N	4°22'56.07"E	YES	YES	YES	YES
Port Napoléon	43°22'38.23"N	4°49'21.11"E	NO	YES	YES	YES
Roustan	43°21'35.05"N	4°50'54.17"E	NO	NO	YES	YES
Saint-Gilles	43°39'58.47"N	4°25'38.16"E	NO	NO	YES	YES
Sites			6	12	18	17
Mosquitoes			4220	5499	7705	4158
Pools			147	1369	257	145
USUV-positive pools			11	1	0	6
WNV-positive pools			-	2	0	0
USUV sequences			YES	YES (1/1)	-	YES (2/6)
WNV sequences			-	YES (1/2)	-	-

All sites were not sampled all years. Thus, a "YES" on a given year indicates sampling in that site and year. Data on the sites from our previous study in 2015 are provided for the sake of completeness. Sites with WNV-positive pools are shown in blue. Sites with USUV-positive pools are shown in yellow. Figures between brackets stand for number of pools yielding a virus sequence among positive pools for a given virus.
